# Assessing the Quality of Mobile Apps Used by Occupational Therapists: Evaluation Using the User Version of the Mobile Application Rating Scale

**DOI:** 10.2196/13019

**Published:** 2019-05-01

**Authors:** Kelsea LeBeau, Lauren G Huey, Mark Hart

**Affiliations:** 1 College of Public Health and Health Professions University of Florida Gainesville, FL United States

**Keywords:** occupational therapy, occupational therapists, mobile apps, mobile applications, technology, computers, handheld, mHealth, mobile health

## Abstract

**Background:**

The continuous development of mobile apps has led to many health care professionals using them in clinical settings; however, little research is available to guide occupational therapists (OTs) in choosing quality apps for use in their respective clinical settings.

**Objective:**

The purpose of this study was to use the user version of the Mobile Application Rating Scale (uMARS) to evaluate the quality of the most frequently noted mobile health (mHealth) apps used by OTs and to demonstrate the utility of the uMARS to assess the quality of mHealth apps.

**Methods:**

A previous study surveying OTs’ use of apps in therapy compiled a list of apps frequently noted. A total of 25 of these apps were evaluated individually by 2 trained researchers using the uMARS, a simple, multidimensional analysis tool that can be reliably used to evaluate the quality of mHealth apps.

**Results:**

The top 10 apps had a total quality score of 4.3, or higher, out of 5 based on the mean scores of engagement, functionality, and aesthetics. Apps scored highest in functionality and lowest in engagement. Apps noted most frequently were not always high-quality apps; apps noted least frequently were not always low-quality apps.

**Conclusions:**

Determining the effectiveness of using apps in clinical settings must be built upon a foundation of the implementation of high-quality apps. Mobile apps should not be incorporated into clinical settings solely based on frequency of use. The uMARS should be considered as a useful tool for OTs, and other professionals, to determine app quality.

## Introduction

### Background

The field of occupational therapy is continuing to increase throughout the United States. According to the United States Department of Labor Bureau of Labor Statistics [1], there are approximately 130,000 occupational therapists (OTs) working in offices, hospitals, schools, nursing homes, and home health services. The projected percent change in employment from 2016 to 2026 for occupational therapy is expected to be 21%, which is much higher than the average growth rate for all occupations of 7% [1].

Occupational therapy is a client-centered health profession, which aims to facilitate rehabilitation, health, and overall well-being through occupation. OTs are allied health professionals who provide the care and support needed to enable injured, ill, or disabled patients to participate in activities of everyday life. They assist clients across all age groups to promote, develop, recover, improve, and maintain abilities allowing them to engage in the occupations needed for daily living and working. Moreover, OTs work with clients to develop ways in which the occupation or the environment can be modified to better support occupational engagement [2,3].

The world of technology continues to grow exponentially every year, and the use of mobile apps is becoming increasingly commonplace. According to the Pew Research Center, the number of US adults who own a smartphone has increased over the past 5 years from 51% in 2013 to 77% in 2018 [4]. With the continuous growth in smartphone usage, an entire new industry for third-party apps has formed. An “app,” short for application, is defined as a self-contained program for smartphones designed to fulfill a particular purpose [5]. As of March 2017, there were 2.2 million and 2.8 million apps available in the Apple App Store and Google Play Store, respectively [6]. Of these 5 million apps, there are more than 318,000 health-related mobile apps with an estimated 200 health apps being added each day [7]. Given the versatility of apps, possibilities for mobile app integration into the field of occupational therapy continues to expand.

Mobile health (mHealth) is the application of technologies to improve health outcomes [8]. mHealth apps have the potential to be extremely beneficial in multiple health fields for several reasons: (1) They are cost effective, (2) They are accessible at any time, (3) They are convenient to the client, (4) They do not require assistance from health care professionals to operate successfully, and (5) They can allow the user to personalize their experience. In addition, mHealth apps have the potential to increase access to evidence-based health information.

As a result of the rise in everyday mobile technology use, the health care system has seen incredible growth in the use, and integration, of mobile apps for the promotion of health and well-being [9,10]. Although there is room for improvement, occupational therapy is among one of the fields that has begun to slowly integrate technology into its everyday practice. Apps are being used as intervention tools, education aides, and for support purposes [11]. A blog poll taken by the American Occupational Therapy Association (AOTA) indicated more than half of practitioners use apps at least occasionally in the clinic [12]. Many websites, including the AOTA website, provide lists of popular apps being used in occupational therapy.

OTs use apps as therapeutic tools inside and outside the clinical setting. Apps can be used to address problems in patients with developmental disorders, traumatic brain injury, stroke, and spinal cord injury. Some therapeutic apps address improving handwriting, fine motor dexterity, motor planning, sequencing, memory recall, social interaction, visual perception skills, and more [11]. Due to the constant development of apps, OTs need to be aware of the quality, efficacy, reliability, and security of apps they are using to ensure best practices and results [13]. It is especially important to consider the reliability and consistency of apps being used, both for clinical and evaluation purposes, as well as the safeguarding of data.

Scales classifying, and rating, the quality of apps are crucial to pinpoint top apps that should be promoted. The 5-star rating scale seen in many app stores has not been empirically proven to enable a potential user to decipher the quality of 1 app versus another [10]. The majority of rating scales aim to understand the user’s perception of the app leading to subjective reviews and selection of apps based on popularity as opposed to quality [10]. Scales need to focus more on classifying the quality of the app and less on rating the developer’s description. Therefore, researchers must take the time to download the app, familiarize themselves with its features, and rate the app accordingly using empirically supported evaluation tools.

Several studies have been conducted regarding mobile device use and decision making, mobile apps use, decision making, and opinions, and technology acceptance and utilization among OTs. Kelly Erickson [14] performed a systematic review of the literature specifically looking at the use of mobile app–based devices in occupational therapy practice. Erickson found limited research evidence related to the use of apps in practice. A total of 3 areas of focus for practitioners were established from the literature review: (1) The mobile app–based devices should be client centered, (2) The role of the OT is to show an individual the possibilities of mobile app–based devices, and (3) The OT should consider features of the mobile app–based device and the chosen apps. Erickson touches on the importance of choosing quality apps; however, the study does not specifically examine quality criteria for apps being chosen by OTs for their clients.

A study conducted by Ravenek and Alvarez [15] developed detailed considerations that can be used to inform OTs’ decisions related to selecting appropriate apps for clinical practice. The considerations proposed allow OTs to weigh therapist, client, and app characteristics so that a specific app can be chosen for a specific client. Although these considerations are valuable for all OTs and important to consider for each client, the study does not discuss how OTs can objectively evaluate the quality of the apps they are individually choosing for their clients.

### Objective

There is a paucity of knowledge surrounding OTs and their use of quality apps in the clinical setting. Although therapists do use their clinical judgement when using apps in therapy settings, such as target skills and preferred features, the objective quality of apps is still unknown [11]. Therapists are likely to achieve better results with clients if they choose to use quality apps; therefore, it is crucial for therapists to be able to distinguish low-quality apps from high-quality apps. To do so, OTs must be aware of resources available to assist them in evaluating apps they frequently use in their practices. The purpose of this study was to use the user version of the Mobile Application Rating Scale (uMARS) to investigate the quality of apps most frequently used by OTs and demonstrate the utility of the uMARS to assess the quality of mHealth apps.

## Methods

### Theoretical Perspective

Before mobile apps are adopted into clinical settings, they first should be evaluated to assess their overall quality. Doing so could help ensure high-quality apps are being used by practitioners and could eliminate some of the cost and time demands associated with the trial and error process of finding apps to use in therapeutic settings. However, there is limited information to guide evaluation of the quality of mHealth apps, and the evaluation tools that are available may not be well known or widely used by occupational therapy practitioners. To facilitate the use of mHealth app quality evaluation tools and the adoption of high-quality apps in therapeutic settings, a diffusion theory approach might be useful.

The diffusion of innovations theory is concerned with how ideas and practices are adopted over time through both formal and informal communication channels and processes. More specifically, diffusion theory can be used to explain how technology spreads as well as the rate at which technology is adopted by its intended audiences in higher education and clinical environments [16,17]. The use of mobile apps is still relatively new within the field of occupational therapy; therefore, not all therapists are willing to adopt and embrace this technology [15]. It is also possible there is a fair amount of uncertainty (a barrier to adopting an innovation) surrounding mHealth apps given the large number of mHealth apps currently available and the limited number of reliable app-quality assessment tools. According to diffusion of innovations theory, the rate of adoption of innovations is largely influenced by 5 innovation characteristics: (1) Relative advantage, (2) Compatibility, (3) Complexity, (4) Trialability, and (5) Observability [16,17]. By determining the quality and effectiveness of mobile apps with a reliable assessment tool, each of these characteristics can be targeted to resolve uncertainties and help facilitate the adoption of quality mobile app use in clinical settings through empirical evidence and informed decision making by OTs.

Diffusion theory also emphasizes the importance of a 2-stage communication approach. That is, information about an innovation “communicated to influential others, friends, relatives, opinion leaders, in the recognition that adoption can be influenced indirectly in this manner” [16,17]. Opinion leaders, also known as early adopters, are well respected and admired by others in their social network and generally possess a large amount of social influence. They are particularly important to the diffusion process because their opinions and adoption of certain behaviors influences the opinions and adoption of certain behaviors of others. Thus, opinion leaders play a crucial role in promoting adoption. It is important to consider the influence of such opinion leaders in the occupational therapy clinical setting and the impact they can have on the adoption of using a tool such as the uMARS to identify high-quality mobile apps. If the usefulness of the uMARS in identifying high-quality apps can be demonstrated to occupational therapy opinion leaders, it is likely they might adopt this innovation. In line with diffusion theory, their adoption of the uMARS might influence the adoption of the uMARS by other OTs [16,17].

Before development of the Mobile Application Rating Scale (MARS), few health-related app-quality assessment tools existed beyond the 5-star rating scale seen in application stores. Although the US Food and Drug Administration (FDA) does provide guidance regarding the development and use of mobile medical apps, as well as considerations for practitioners and clients, the FDA does not specifically provide a user-friendly assessment rating tool. Other organizations, such as the American Medical Association and the Healthcare Information and Management Systems Society, have developed mHealth app guidelines. There is no consensus, however, about which apps are the best to use or the highest quality. Some countries have developed systems or processes for health apps to be assesses for safety and/or quality. For example, Spain has the AppSoludable Quality Seal and the United Kingdom has their NHS Digital Apps Library.

### Mobile Application Rating Scale and User Version of Mobile Application Rating Scale

The initial goal of the MARS was to create a tool that trained researchers could utilize to determine whether mHealth apps satisfied certain quality criteria instead of relying on the subjective 5-star rating system. Thus, the MARS was created as one of the first reliable and objective instruments for trialing, classifying, and rating the quality of mHealth apps [10]. The MARS provides a multidimensional measure of 4 objective quality app indicators: engagement, functionality, aesthetics, and information quality. It also includes a subject quality indicator. In addition to being easy-to-use, the MARS is widely applicable to various health domains and can be modified to measure the quality of apps with no relation to health. The MARS has demonstrated excellent internal consistency, interrater reliability, and validity [10].

Adapted from the 23-item MARS rating tool, the uMARS was developed as a simpler, more user-friendly alternative to the MARS tool. The MARS requires training and expertise in mHealth and the relevant health field to be administered [10]. The uMARS eliminates the need for trained experts and provides a reliable tool to assist app developers and researchers with assessing the quality of mHealth apps [10,13]. The scale consists of a 20-item measure including 4 objective quality subscales: engagement, functionality, aesthetics, and information quality. In addition, 1 subjective quality subscale and 1 6-item perceived impact subscale is included. The uMARS has good reliability, proven through test-retest studies, and excellent internal consistency (full scale Cronbach alpha=.90), with high individual alphas for all subscales [13]. The subjective subscales of the uMARS also have very high internal consistencies, with an engagement alpha of .80, a functionality alpha of .70, an aesthetics alpha of .71, and an information alpha of .78 [13]. The reliability for each subscale was highest for engagement, functionality, and aesthetics [13]. This indicates the uMARS provides an accurate measure of app quality for target users.

### Data Collection

A total of 30 mobile apps were selected for evaluation. Mobile apps were initially chosen based on their frequency noted in a peer-reviewed research study performed by Seifert et al (2017) titled, “Apps in therapy: OTs’ use and opinions” [11]. The cut-off for frequency noted was 4; therefore, OTs surveyed in the mentioned study [11] must have noted use of the mobile app 4 or more times for it to be included for evaluation. Additional inclusion criteria for app selection included the app was in English, available through the US Apple App store, compatible with iPad, and US $9.99 or under. A total of 5 apps were excluded because they did not meet these additional inclusion criteria. Thus, a total of 25 apps were selected for review. The 25 apps were reviewed by 2 trained uMARS evaluators. All apps were reviewed and evaluated on iPads because these are often the mobile devices used in clinical settings.

The principal researcher and another recruited student from the University of Florida evaluated each app (N=25) using the uMARS tool. The student evaluator was selected through convenience sampling. Both researchers of this study had previously attended a uMARS training session facilitated by researchers from a different study at the University of Florida. During the session, the trainees watched 3 video tutorials detailing the procedure for evaluating apps using the uMARS. During the training session, the researchers reviewed the uMARS rating tool and evaluated 2 trial apps to demonstrate appropriate mastery of the uMARS. Each trial app was examined for 10 min and then independently rated by the trainees. The training session lasted 60 min.

Upon completion of the training session, the principal researcher distributed the list of 25 mobile apps to the other evaluator. All 25 apps were then individually assessed by both evaluators according to 3 validated subscales on the uMARS: (A) Engagement, (B) Functionality, and (C) Aesthetics. These 3 subscales were chosen because of their internal consistencies and their test-retest reliabilities [13]. There was also a system in place in case discrepancies occurred among reviewers. To enhance reliability of evaluators’ scores, both reviewers evaluated the apps separately then came together to discuss scores. Any discrepancies were then discussed until an agreement was made between the reviewers. In a study with more than 100 apps, inter-rater reliability is, and should be, measured.

### Data Analysis

The uMARS rating tool was used to evaluate the quality of mobile apps most frequently used by OTs. Apps were rated using iPads, as they are the most common mobile device used in clinical settings. To collect and analyze descriptive and technical information about each app, 3 of the uMARS subscales were used: (A) Engagement, (B) Functionality, and (C) Aesthetics. For uMARS sections A, B, and C, items are rated on a 5-point scale (1-inadequate, 2-poor, 3-acceptable, 4-good, and 5-excellent). This study was performed to understand how user-friendly the uMARS could be for OTs (and the broader health professionals). This was based on the validated measurements of subscales A, B, and C, as explained in the previous section [13]. The researchers chose not to include the subjective measures of the uMARS. However, these are options for users to rate if they so desire.

The individual scores for each section were determined by calculating the mean of the ratings for each question in that designated section. This calculation provided the top ranked apps in each section. The total quality score of the app was determined by averaging the mean scores of the engagement, functionality, and aesthetics sections. This calculation provided the highest scoring apps overall.

## Results

### App Inclusion

A total of 30 mobile apps were considered at the start of this study. On the basis of inclusion criteria established by the researchers, 5 mobile apps were not included. Thus, a total of 25 mobile apps were evaluated using the uMARS for this study. Scores for engagement, functionality, and aesthetics were calculated using the 5-point scale. The scores described in the upcoming sections are somewhat high. As a reminder, these apps were already prescreened as apps used by the OT population [11]. It is likely these apps were of higher quality to begin with, which would help explain the high scores.

### Engagement

Engagement criteria were evaluated based on entertainment, interest, customization, interactivity, and target group appeal. Entertainment means how fun and entertaining the app is to use and how the components making the app fun compare with similar apps. Interest means how interesting the app is to use and how the information is presented compared with similar apps. Customization means whether the app allows the user to customize settings and preferences such as sound, content, and notifications. Interactivity means whether the app allows user input, provides feedback, and contains prompts such as reminders, sharing options, and notifications. Target group appeal means whether the content (visuals, language, and design) is appropriate for the target audience.

Averages for engagement were calculated for all apps included in the study. The 10 apps with the highest average mean scores in terms of engagement can be found in Table 1. Fit Brains scored a perfect 5 for engagement. Fit Brains is highly entertaining and interesting to use, would stimulate repeated use, allows for the user to tailor all preferences and settings, has a high level of responsiveness through interactive features and feedback, and is designed specifically for its target audience. Apps scoring close to 5, Lumosity and Bugs & Buttons, demonstrated many of the same qualities previously mentioned.

### Functionality

Functionality criteria were evaluated based on app performance, ease of use, navigation, and gestural design. Performance means how accurately and quickly the app features (functions) and components (buttons, menu) work. Ease of use means how easy it is to learn how to use the app and how clear the menu labels, icons, and instructions are to the user. Navigation means how logical the flow and movement between screens is for users and whether the app has all the necessary links to navigate between screens. Gestural design means the taps, swipes, pinches, and scrolls make sense to the user and are consistent across all components and screens. The 10 apps with the highest average mean scores in terms of functionality can be found in Table 2.

**Table 1:** Top 10 averaged scores of occupational therapy apps evaluated in section A: engagement of the user version of the Mobile Application Rating Scale evaluation.

Occupational therapy app title	Average score
Fit Brains	5
Lumosity	4.7
Bugs & Buttons	4.4
Bugs & Bubbles	4.3
Writing Wizard	4.2
Ready to Print	4.1
Peekaboo Barn	4.1
Choice Works	4.1
Visual Attention	3.9
Handwriting Without Tears Wet, Dry, Try	3.8

**Table 2:** Top 10 averaged scores of occupational therapy apps evaluated in section B: functionality on the user version of the Mobile Application Rating Scale evaluation.

Occupational therapy app title	Average score
Bugs & Buttons	5
Ready to Print	5
Writing Wizard	5
Bugs & Bubbles	5
Handwriting Without Tears Wet, Dry, Try	5
Letter School	4.875
Letter Reflex	4.75
Cursive Writing Handwriting Without Tears HD Style	4.75
Fit Brains	4.75
Toca Kitchen	4.75

The mean scores for functionality were calculated to determine the top 10 apps in this category (Table 2). A total of 5 of the apps scored a perfect 5 (Bugs & Buttons, Ready to Print, Writing Wizard, Bugs & Bubbles, and Handwriting Without Tears [HWT] Wet, Dry, Try), which indicates a highly functional app according to uMARS criteria. The functionality category had the highest overall scores on the uMARS. The average of all 25 apps was 4.535, and none of the apps evaluated scored below a 3.625. This indicates a majority of the apps have fast performance features; have little or no technical bugs; are simple, intuitive, and easy to use immediately without instructions; have logical, easy, and clear screen flow; and have consistent gestural designs.

### Aesthetics

Aesthetics of apps were evaluated based on layout, graphics, and visual appeal. Layout means how appropriate the arrangement and size of buttons, icons, menus, and content on the screen are for the user. Graphics means how high the quality and resolution of the graphics for buttons, icons, menus, and content appear in the app. Visual appeal is described as how good the app looks, as well as the overall stylistic consistency of the app. The 10 apps with the highest average mean scores in terms of aesthetics can be found in Table 3.

Over half (64%) of the 25 apps scored over a 4 on aesthetics. As seen in Table 3, 3 apps scored a perfect 5 (Bugs & Buttons, Bugs & Bubbles, and Toca Kitchen). These 3 apps had appropriate layouts with professional, simple, clear, orderly, and logically organized designs; very high-quality graphics, visual designs, and resolutions; and very attractive, memorable, and outstanding visual appeal. The lowest scoring app, Visual Timer, scored a 3 because of satisfactory layout; few problems selecting, locating, seeing, and reading items; moderate-quality graphic and visual design; and average visual appeal, which is neither pleasant nor unpleasant.

**Table 3:** Top 10 averaged scores of occupational therapy apps evaluated in section C: aesthetics on the user version of the Mobile Application Rating Scale evaluation.

Occupational therapy app title	Average score
Bugs & Buttons	5
Bugs & Bubbles	5
Toca Kitchen	5
Lumosity	4.83
Peekaboo Barn	4.83
Fit Brains	4.66
Letter School	4.5
Cursive Writing Handwriting Without Tears HD Style	4.46
Cursive Touch & Right	4.3
Write My Name	4.3

**Table 4:** Top 10 occupational therapy apps based on the overall average score from the user version of the Mobile Application Rating Scale evaluation.

Occupational therapy app title	Average score
Fit Brains	4.803
Bugs & Buttons	4.8
Bugs & Bubbles	4.766
Lumosity	4.718
Toca Kitchen	4.483
Writing Wizard	4.45
Peekaboo Barn	4.435
Ready to Print	4.366
Letter School	4.325
Handwriting Without Tears Wet, Dry, Try	4.31

### Top Occupational Therapy Apps

The top 10 occupational therapy apps were chosen according to their average scores derived from the uMARS subscales of engagement, functionality, and aesthetics, each with its own independent validity (Table 4). A 5-point rating scale (1-inadequate, 2-poor, 3-acceptable, 4-good, 5-excellent) was utilized for each subscale, and then all 3 subscales were averaged to determine the final score for each app. Many of the highest scoring apps overall appeared on the top 10 lists of apps for each individual uMARS category.

The top apps all scored above a 4.3, with only a small amount of variance between each one. These apps are geared toward improving fine motor skills, spatial reasoning skills, or cognitive functioning skills. Overall, the apps scoring highest in functionality often scored lowest in engagement. In addition, 4 out of the 10 apps (Writing Wizard, Ready to Print, Letter School, and HWT Wet, Dry, Try) are all focused solely on improving handwriting.

Fit Brains was the highest scoring app overall with a uMARS score of 4.803 (Table 4). Fit Brains had high scores in all 3 categories, indicating a high-quality app according to the uMARS rating tool. The lowest scoring app overall had a uMARS score of 3.33. Out of the 25 apps scored for this study, all of their overall scores ranged from 3.33 to 4.03, meaning most of these apps have a relatively average or above average score.

## Discussion

### Principal Findings

The uMARS is a simple tool that can easily be used by end users to evaluate the quality of mHealth apps, including end users in clinical settings such as OTs. The uMARS utilizes multidimensional analyses to measure certain qualities of mobile apps, including engagement, functionality, and aesthetics. According to the study performed by Stoyanov et al [9], the uMARS has good internal consistency (alpha=.90) and high inter-rater reliability, thus indicating the uMARS is a reliable tool for quality ratings of apps used by OTs.

The purpose of this study was to evaluate the quality of the most frequently noted mobile apps used by OTs based on a previous study that surveyed OTs most frequently used mobile apps in therapy [11], as well as demonstrate the utility of the uMARS to assess the quality of mHealth apps. The results of this study indicate mobile apps should not be incorporated into clinical settings solely based on frequency of use by OTs. Many of the apps analyzed in this study were not necessarily high-quality apps according to the uMARS. In the same way, many of the apps noted least frequently were not necessarily low-quality apps according to the uMARS analysis tool. The results also show how the uMARS can be used to score the quality of mHealth apps in an objective manner.

Multimedia Appendix 1 shows the comparison between apps most frequently used by OTs [11] and the uMARS scores of 25 of those most frequently used apps. The apps (middle column) in Multimedia Appendix 1 are presented in order of frequency (left column) with their respective uMARS score and rank (right column). On the basis of analyses performed in this study, there is no clear relationship between apps being noted more frequently and the quality of those apps being higher. Letter School, an app noted by OTs 69 times in the study by Seifort et al, scored an overall 4.325 and ranked 9th out of 25 apps. The overall highest scoring app on the uMARS, Fit Brains was only noted by OTs 5 times in the study by Seifert et al [11]. The lowest scoring app on the uMARS, however, was noted 6 times. Therefore, it seems there is an apparent disconnect between high-quality apps and their usage by OTs. It is imperative OTs utilize more appropriate quality measurements to determine which apps are best to use in a clinical setting. The uMARS offers a quick and easy way for OTs to measure such quality and select high-quality apps because just as simplicity is important so is a tool that requires little time to use.

### Limitations

This study is not without limitations. As is the nature of mobile apps and mobile technology, apps and technologies are regularly undergoing changes and updates. Apps in the Apple App store are no exception. Since conducting the uMARS evaluation, it is possible many of the apps reviewed have been updated to newer versions. The updates on the most up-to-date versions could alter the results of this analysis. New features could have been added, aesthetic elements could have been changed, and glitches could have been fixed. This study also did not compare the market rating (5-star app store rating) with the quality scores from the uMARS. This information could provide important information about the accuracy and trustworthiness of app store ratings. Future research could be conducted comparing the market rating with an app’s uMARS score. Moreover, new mobile apps could have been developed since the compilation of the apps reportedly used by OTs [11]. Updates and changes to mobile apps and technologies must be kept in mind when performing studies such as this one. If OTs are choosing to utilize mobile app technology in their clinical settings, however, it is also their responsibility to attempt to keep up with the ever-changing mobile app industry. Future research should build off of this study by using the uMARS tool to analyze the quality of new and improved mobile apps. This study only used 3 subscales of the uMARS tool to evaluate mobile apps. To add to this area of research, the uMARS tool in its entirety should be used to evaluate mHealth apps. There are also other considerations that go into incorporating mHealth apps into a clinical setting. This study looked at 1 aspect of these considerations (the quality of mHealth apps being used); however, it is important to note other aspects of mHealth apps should be considered. One aspect of importance is the security of data collected within mHealth apps. This is a critical feature not specifically measured through the uMARS but that must be considered when using mHealth apps in clinical settings.

The list of apps used in this study came from a previous study that surveyed 20 OTs in Ohio [11]. The majority (40.5%) of these OTs worked in pediatric settings. This resulted in a majority of the reported apps being used for a patient population aged younger than 12 years. Therefore, the results of this study may not be generalizable to mobile apps used by OT populations outside of pediatric settings. To address this limitation, future research should be expanded to include OTs and mobile apps from a variety of settings. The AOTA website offers a comprehensive list of apps for occupational therapy practitioners [12]. Although not quality reviewed, this might be a good place to start.

### Conclusions

As the use, and introduction, of mobile apps continues to grow, it will become increasingly important for therapists to adopt high-quality apps. Consistent with diffusion theory, by determining the quality and effectiveness of mobile apps with a reliable assessment tool such as the uMARS, the 5 attributes of innovation (relative advantage, compatibility, complexity, trialability, and observability) can be targeted to help facilitate widespread adoption of quality mobile app use in occupational therapy clinical settings [16,17]. To promote the use of the uMARS tool in clinical settings, the influences of opinion leaders (eg, early adopters) and their respective social networks should be considered [16,17]. By encouraging OT opinion leaders to adopt the use of the uMARS in their clinical setting, it is likely these influencers will intentionally or inadvertently influence other OTs to also adopt the use of the uMARS [16,17].

Future research can focus on the top-rated apps found in this study to determine effectiveness in therapy. It is important to understand the implications a high-quality app can have as a complement to the occupational therapy services provided to a patient. Investigating whether or not utilizing mobile apps in clinical settings is helpful to the overall rehabilitation of a patient is crucial. These apps could also be compared in different settings to examine if some mobile apps are best suited for 1 setting instead of another. In addition, it is important to determine if using higher rated apps results in increased occupational therapy gains. As the overall goal is to benefit the OTs and the patients receiving therapy, future research is needed to understand if the quality of the app is directly related to how well a patient does in therapy. The evidence supporting the effectiveness of using higher quality apps has been addressed minimally in research. Understanding the effectiveness of high-quality apps compared with low-quality apps could inform practitioner’s decisions about using mHealth apps in therapy. Furthermore, qualities of apps that ranked higher could be used to improve existing apps or help with the development of new ones. As apps in this study scored lowest in engagement, focusing on improving engagement among existing apps could be a good place to start. Future apps could learn from this research by ensuring user engagement is prioritized during the development phase. In addition, this study can be used as a guide for using uMARS for future evaluations of apps utilized in occupational therapy clinical settings. In this way, OTs will have access to a validated tool to help them choose high-quality apps to use for their specific patient population.

## Appendix

Multimedia Appendix 1 Comparison of the top 25 most frequently noted apps by occupational therapists with their total user version of the Mobile Application Rating Scale (uMARS) score and respective rank based on uMARS score.

## Abbreviations

AOTA: American Occupational Therapy Association
HWT: Handwriting Without Tears
MARS: Mobile Application Rating Scale
mHealth: mobile health
OT: occupational therapist
uMARS: user version of the Mobile Application Rating Scale
