# Supplementary material for: Assessing the Quality of Mobile Apps Used by Occupational Therapists: Evaluation Using the User Version of the Mobile Application Rating Scale
Source: JMIR Mhealth Uhealth. 2019 May 1;7(5):e13019. doi: 10.2196/13019 (PMC6526689; doi:10.2196/13019)
Supplement: Multimedia Appendix 1 [file mhealth_v7i5e13019_app1.pdf]

Multimedia Appendix

Top 25 apps listed by frequency noted by OTs compared to their total uMARS score and respective uMARS rank.

**Frequency Noted**	**App Title**	**Total uMARS Score (*Rank*)**
69	Letter School	4.325 (*9*)
48	Dexteria	3.858 (*21*)
17	Bugs & Buttons	4.8 (*2*)
16	Ready to Print	4.366 (*8*)
16	Writing Wizard	4.45 (*6*)
9	Lumosity	4.728 (*4*)
8	Letter Reflex	3.626 (*22*)
7	Dragon Dictate	3.55 (*23*)
7	Cursive Writing Handwriting Without Tears HD Style	3.97 (*18*)
7	iWriteWords	4.005 (*14*)
7	Cursive Touch & Right	4.108 (*11*)
7	Zones of Regulation	3.908 (*20*)
6	Bugs & Bubbles	4.766 (*3*)
6	Doodle Buddy	3.33 (*25*)
6	Flow	3.99 (*17*)
6	Matrix Game 1	3.95 (*19*)
6	Peekaboo Barn	4.435 (*7*)
6	Visual Timer	3.483 (*24*)
5	Fit Brains	4.803 (*1*)
5	Toca Kitchen	4.483 (*5*)
5	Visual Attention	4.033 (*13*)
5	Write My Name	4 (*15*)
4	Choice Works	4.06 (*12*)
4	Handwriting Without Tears Wet, Dry, Try	4.31 (*10*)
4	Start Dot Handwriting	3.991 (*16*)
